# Microplastic-mediated delivery of di-butyl phthalate alters C. elegans lifespan and reproductive fidelity

**DOI:** 10.21203/rs.3.rs-6629868/v1

**Published:** 2025-05-21

**Authors:** Chiara Angelyn O. Maldonado, David M. Mares, Paola C. Garcia, Maria F. Gamez, Midori R. Flores, Alyssa D. Friudenberg, Ryan L. Peterson, Jennifer C. Harr

## Abstract

Microplastics harbor chemical additives and absorb pollutants from the environment. Microplastics pose a human health threat and have been found in nearly all human tissues. The toxicological pathways and physiological effects of microplastic-mediated chemical exposure following ingestion remain unknown. Here we use *C. elegans* to investigate the effects of di-butyl phthalate and polystyrene microplastic mixtures on fertility and lifespan. Our studies demonstrate that 1 µm microplastics at 1 mg/L exposure levels result in decreased brood size, whereas 1000 times fewer microplastics (1 µg/L) did not affect the number of eggs laid. While there was no change in brood size at 1 µg/L microplastic exposure levels, there was an increase in embryonic lethality. Microplastics-mediated delivery of di-butyl phthalate to *C. elegans* significantly reduced brood size and increased embryonic lethality compared to exposure to microplastics alone. This reproductive toxicity is potentially due to a stress response via DAF-16, as observed with microplastics and di-butyl phthalate co-exposure. Furthermore, chronic exposure to microplastics shortened the lifespan of *C. elegans* , which was further reduced with di-butyl phthalate co-exposure. The exacerbated defects observed with co-exposure to phthalate-containing microplastics underscore the risks associated with microplastics releasing the additives and/or chemicals that they have absorbed from the environment.

## Introduction

Plastic and chemical pollutants are ubiquitous in the environment, posing an emerging human health concern. Microplastic (MP) particles have been identified in nearly all human tissues, from blood[1] to the brain[2], and most recently in human reproductive organs[3,4]. Microplastic exposure includes plastics from various environmental sources and exists in a wide range of chemical types, mixtures, and conditions. Primary microplastic particles are manufactured and used as ingredients in biomedical products and various health and beauty items (i.e., scrubs, make-up, cleansers, and toothpaste)[5,6]. Whereas secondary microplastics result from the breakdown of larger plastic items. Human exposure to microplastics occurs through direct use of microplastic-containing products, exposure to secondary microplastics released into food, or environmental pollutants.

Both primary and secondary microplastics undergo chemical and physical changes in their structure due to environmental exposure. Factors such as UV light, physical breakdown, and exposure to environmental pollutants can lead to alterations in the size, surface structure, and chemical composition of microplastics. Despite efforts to reduce plastic use, it remains prevalent, with much already having entered the ecosystem[7]. Dating back to the 1950s, approximately 400 million tons of plastic waste accumulate each year[8]. Microplastics (MPs, plastic particles < 5 mm[9]) and nanoplastics (NPs, plastic particles ≤ 1 µm[9]) are detected in the air, water, and our food sources[10–14]. Meanwhile, microplastics have been found in tissue exhibiting the ability to enter mammalian cells[15]. The smallest particles, nanoplastics, invade endosomes, lysosomes, lymph and circulatory systems, and the lungs[16–18], leading to deleterious effects on the cellular level[17,18]. The direct health risks of human ingestion of microplastics have not been quantified. Moreover, these risks amplify as plastics already contain additives, and they can absorb, accumulate, and transfer chemicals from the surrounding environment into organisms.

Plastic and resulting microplastic particles can be composed of homogenous or heterogeneous polymer mixtures, contain additives from the manufacturing process (plasticizers, by-products, and monomers), and may absorb chemical pollutants[19–21]. These additives and pollutants can later leach into the environment[22]. Persistent organic pollutants have been shown to be transferred from plastic particles to fish with adverse effects in environmentally relevant concentrations[23] and led to a transfer of chemicals from microplastics into the guts of lung worms (*Arenicola marina*), and a reduction of biological functions[24]. In mice, it was demonstrated that phthalate esters were released from microplastics leading to intestinal permeability and inflammation[25]. A study using *C. elegans* showed that 19 chemicals (including phthalates and agrochemicals) increased DNA damage and physiological dysregulation when compared to Bisphenol A (BPA)[26]. BPA has been widely recognized as a threat to human health, and its use has been highly restricted, with a total ban on its use in infant-related products[27,28]. Due to these concerns, most manufacturers have voluntarily removed BPA from consumer products. With these regulations and demands from consumers, manufacturers must rely on other plasticizers to aid in the performance and functionality of plastic products.

Di-butyl phthalate (DBP), a widely used plasticizer, can compose 20–80% of some plastic products[21] and its use is regulated by both US and European agencies[29]. DBP is classified as a phthalate esters, which as a group, are considered endocrine-disrupting chemicals and pose cytotoxic and estrogenic effects[28,30–32]. DBP is most commonly used as a plasticizer, primarily in polyvinyl acetate emulsion adhesives, as a solvent for oil-based dyes, and insecticides[33]. It can be found in consumer products including nail polish, paints, adhesives, and perfume oils. Prior to 2008, many products for children contained high levels of DBP, butyl benzyl phthalate (BBP), and di (2-ethyexyl) phthalate (DEHP). The United States and European federal restrictions now limit the use of some phthalates, including DBP, in children's products. Despite bans on some products, DBP and other phthalate esters are widely used in everyday household items or manufacturing processes. Many household products contain these chemical additives, which, when disposed of, will likely end up in municipal landfills[33]. Thus, DBP could leach into the environment due to the improper disposal and breakdown of products containing it. Once in the environment, DBP absorbs onto suspended particles and is less likely to degrade than dissolved DBP[33]. DBP has a high likelihood of being absorbed into MPs in the environment as it is readily absorbed by plastic particles, possesses properties that make it absorptive, and it is chemically related to compounds that are easily absorbed into plastics[34].

Despite the risks of microplastic exposure to humans and the danger of plastic particles carrying chemical pollutants, microplastics remain under-investigated. It is crucial to determine how microplastic-chemical mixtures alter the development and health of humans and other land animals. We use the *C. elegans* model system to examine the cellular and physiological effects of nanoplastic mixtures on whole-organism health and development. In this study, we investigate whether 1 µm polystyrene microplastics can transport absorbed chemicals into an animal after ingestion and lead to physiological responses. The 1 µM polystyrene size regime is ideally situated at the interface of micro- and nano-particles, and because polystyrene is commonly used in packing foams, food containers, and disposable cutlery, it is highly relevant to human oral microplastic exposure risks[21]. The additive, DBP, was chosen as a representative chemical due to its wide use as an industrial additive. Studies of additives commonly found in plastics show that the many common plasticizers with relatively low molecular weight will transfer from the plastic material into foods[21]. We hypothesized that microplastics can contain a chemical pollutant and mediate physiological defects that are greater than either pollutant alone. We find that microplastic exposure reduces brood size and leads to embryonic lethality in a concentration-dependent manner. The microplastic-mediated delivery further decreased reproduction compared to microplastics or DBP alone. Reproductive defects due to microplastic -DBP co-exposure appear to cause stress via DAF-16 and reduce *C. elegans* lifespan.

## Results and Discussion

*Polystyrene microplastic surface structure is unchanged after exposure to di-butyl phthalate.*

To verify the diameter and surface structure characteristics of polystyrene microplastics (PS MP, hereon referred to as microplastics), scanning electron microscopy (SEM) was used to image microplastics with and without exposure for 24 hours to 0.1 M di-butyl phthalate (DPB) or the solvent (DMSO). SEM analysis revealed no change to the surface morphology of microplastics exposed to DBP for 24 hours. A small but significant increase in the average size of microplastic particles occurred when soaked in DMSO, but not 0.1 M DBP (Fig. 1, $p \leq 0.0001$). It was anticipated that exposure to the plasticizer DBP may change the size or surface structure of microplastics. Environmental exposure of microplastics to heat, UV and mechanical stress can change their characteristics[20,35], and this was not observed after 24 hour exposure of microplastics to DBP or DMSO (Fig. 1). DBP has high rates of sorption into microplastics, with highest rates seen in polystyrene (PS) microplastic when compared to polyvinyl chloride (PVC) and polyethylene (PE)[34]. Sorption of compounds on or into microplastics is size dependent, with smaller particles containing more readily available chemicals, due to the increase in surface to size ratio that occurs with the reduced particle size[20,36,37]. Microplastic and DBP mixtures were prepared by soaking microplastics in DBP for 24 hours, followed by dilution into *C. elegans* food, *E. coli* OP50. In each dilution step, microplastics were sonicated to aid in resuspension. This process would disrupt any DBP that would be adsorbed onto the microplastic surface, and thus any DBP present in the microplastic is assumed to be absorbed within the microplastic particles. The slightly larger but non-significant increase in mean size of the 0.1 M DBP soaked microplastics and significantly larger diameter of microplastics after DMSO exposure may be due to swelling of the microplastic particles after absorption.

*Polystyrene microplastics are ingested by C. elegans and act as a vehicle for chemical exposure.*

*C. elegans* exposed to 1 μg/L and 1 mg/L Dragon-Green microplastic show the presence of microplastics in their gut tube (Fig. 2a). Nematodes were initially exposed to two different doses (1 μg/L and 1 mg/L), which were within the range of other studies and that mimic concentrations determined from human samples such as blood[1,38–41]. Microplastics were present at all larval life stages, and the percentage of total worms containing microplastics increased with life stage and exposure time. By adulthood, nearly all worms contained microplastics (Fig. 2c). These results are consistent with other studies demonstrating that microplastics (> 1 μm) accumulate in the gut, while smaller nanoplastic (< 1 μm) particles can cross cell membranes[15,40,42–44]. When examining the eggs of adult nematodes exposed to Dragon-Green microplastic, no microplastics were observed (Fig. 2b). Smaller microplastics, less than 1 μm in size, in the nanometer range, have been observed to cross into the body cavity in *C. elegans*[42].

Microplastics contain chemical additives or may absorb pollutants in the environment that can then be transferred into living organisms, leading to physiological effects[21,34]. Phthalates, including DBP, are classified as endocrine disruptors that can be long-lived in the environment[21,36]. DBP is most commonly used as a plasticizer in PVC followed by PET[21]. While DBP is not commonly used in PS products, we choose to study it as a potential environmental chemical that could be absorbed in microplastics and transferred into an organism after ingestion. To visualize this, we soaked PS microplastics in the lipophilic dye, Nile Red, to ask if microplastics could mediate the delivery of a chemical into an organism. Nematodes exposed to microplastics that have been soaked in Nile Red do not show puncta, indicating the location of each microplastic, but rather there is diffuse staining in the worm (Fig. 3a). This indicates that the Nile Red dye, which had been absorbed into the PS microplastic, transfers into the worm's body after ingestion. This transfer is present in more than 90% of the worms examined (Fig. 3b). When nematodes are exposed to an equivalent amount of Nile Red without microplastics, the Nile Red is not present in the worm's body. Thus, the microplastics mediate the delivery of Nile Red, which is then absorbed. This mimics what one would expect if microplastics can act as a vector to deliver chemicals into an organism after ingestion.

*Exposure to polystyrene microplastics and polystyrene microplastic DBP mixtures results in reproductive toxicity.*

To determine if microplastics and chemical pollutants cause reproductive toxicity, *C. elegans* were exposed to different concentrations of microplastics and DBP for varying times. When L1 life stage, nematodes were exposed to 1 μm MP (at 1 μg/L concentration) for 48 hours, until the young adult stage, or continuously, there was no observed significance in decreased total eggs laid or hatched (Fig. 4a, S1a). Despite no change to the number of eggs laid, chronic microplastic exposure to nematodes significantly increased embryonic lethality ($p = 0.04$) and the total number of eggs unhatched per nematode ($p = 0.007$) (Fig. 4b, S1b). Embryonic lethality is determined by taking the total number of eggs that are unhatched and dividing by the total number of eggs laid per worm. These results are in range with other studies that observed continuous microplastics of the same or smaller size leading to minimal or no change in brood size, with no embryonic lethality[44]. While some studies noted a decrease in brood size with exposure to microplastics, this difference may be due to a different size or composition of microplastics or varying exposure conditions[45]. When we exposed *C. elegans* to a 1000x higher concentration of microplastics (1 mg/L), we observed a significant reduction in the total number of eggs laid ($p = 0.016$), but this did not lead to an increase in embryonic lethality (Fig. 4a,b).

*C. elegans* exposure to microplastics (1 µg/L) harboring 0.1 M DBP, further reduced the number of eggs laid per worm compared to exposure to microplastics alone ($p = 0.017$, Fig. 4c). This combined exposure did not lead to an increase in embryonic lethality (Fig. 4d). Examination of microplastics loaded with either 100 µM or 3.7 mM DBP did not affect brood size, egg hatching, or embryonic lethality (Fig. 4e,f and S2 a-f). Typical concentrations of DBP used as a plasticizer can range from 10–35%, or higher in some products[21]. Our study employed 0.1 M DBP, a concentration equivalent to less than 10% w/w, which is in the range of DBP found in everyday products. While 0.1 M DBP led to defects in fertility, these were not seen with lower concentrations (100 µM and 3.7 mM). Comparable levels of chemical pollutants could reach organisms through the ingestion of microplastics that have absorbed chemicals from the environment or by the leaching of additives from microplastics that initially contained higher chemical amounts. In particular, it is worth noting that recycled plastics can contain additives from recycled feedstocks[46].

Impaired reproduction with exposure to microplastics alone or in combination with DBP could be caused by various factors. DBP is an endocrine disrupting chemical and leads to cryptogenic effects[32]. Exposure to DBP leads to defects in reproduction and development[29]. Specifically, DBP exposure reduced human sperm function and in rodent studies defects occur in both development and reproduction[47–50]. In *C. elegans*, exposure to 100 µM DBP, in the absence of MP, led to increased embryonic lethality and DNA damage[26]. *C. elegans* exposure to DBP alone, at a lower dose (500 µM) than used in this study, leads to a reduction in the number of eggs laid and increased embryonic lethality[26]. These defects were attributed to defects in early embryogenesis, with elevated levels of DNA double-strand breaks, activation of a DNA damage checkpoint, and impaired embryogenesis[26]. Errors in cell division could reduce the ability of eggs to hatch and could be caused by a multitude of sources[51]. In humans and other organisms, these errors may lead to aneuploidy, spontaneous abortions, and birth defects[26,51,52]. Additionally, a reduction in the total number of eggs laid could be attributed to a nutrient deficiency of the parent caused by intestinal damage from polystyrene microplastic exposure[45,53–56]. Another study suggested that the associated defects with polystyrene microplastic exposure could be due to a reduction in ATP levels and a reduced energy budget toward reproduction[57].

The exacerbated defects we observed with co-exposure to MPs containing 0.1 M DBP demonstrate that microplastics can mediate the delivery of a chemical into an organism after ingestion. This microplastic-mediated delivery of DBP underscores the risks of co-exposure when microplastics can release the additives they already contain and/or chemicals that they have absorbed from the environment.

*Exposure to polystyrene microplastics and polystyrene microplastic DBP mixtures elicits a stress response.*

A possible mechanism of polystyrene microplastic toxicity resulting in a reduction in fertility is the generation of reactive oxygen species (ROS). Polystyrene microplastic exposure leads to higher levels of reactive oxygen species[16,39,53] and increased expression of GST-4 (glutathione S-transferase 4, an enzyme that is involved in clearing ROS) in nematodes[44,55]. Additionally, DBP and other phthalate esters cause DNA damage and chromosomal abnormalities[26,52,58] that can be caused by ROS generation[52]. In response to stress, *C. elegans* activate a DAF-16 stress response that leads to the activation of genes encoding proteins involved in response to oxidative stress[59]. Therefore, to investigate if stress may be an underlying mechanism of reduced fertility, we asked if microplastic-mediated DBP exposure in *C. elegans* would initiate a DAF-16 stress response. The *C. elegans* strain TJ356 contains a reporter GFP fused to DAF-16 that is driven by the *daf-16* promoter[60]. This GFP reporter is cytoplasmic under normal conditions and relocates to the nucleus to activate genes involved in an anti-oxidative response, under stress. Therefore, the localization of this DAF-16 GFP reporter can be used as a readout of a DAF-16-mediated stress response[39,44,61].

Under control conditions, when *C. elegans* were not exposed to microplastics, the majority of worms examined had cytoplasmic DAF-16 localization (Fig. 5a,b). Some worms showed an intermediate localization where less than 30% of the nuclei showed nuclear expression. This same expression pattern is present with *C. elegans* exposure to 1 µg/L microplastics (Fig. 5b). These results are consistent with what others see with similar PS microplastic exposure[44]. In contrast, microplastic-mediated DBP exposure lead to a significant activation of the DAF-16 stress response, with 40% of the total worms showing an intermediate expression pattern and nearly 20% nuclear localization (Fig. 5b). This indicates that the DAF-16 transcription factor is located in the nucleus where it will activate genes in response to oxidative stress. Importantly, exposure to microplastics containing DMSO, the solvent for DBP, or an equivalent amount of DBP or DMSO alone, did not affect DAF-16 localization (Fig. 5b). Expression of DAF-16 in these controls, showed no significant change when compared to the negative control (*E. coli* OP50 only) and microplastic exposure alone (Fig. 5b). In our initial reproductive toxicity studies, we observed that higher concentrations of MPs (1 mg/L) induced a reduction in total eggs laid with no effect on embryonic lethality (Fig. 4a,b). Therefore, we asked if microplastics alone, at a higher concentration, would elicit a stress response via DAF-16. Continuous exposure of *C. elegans* to 1 mg/L microplastics increased the percentage of worms with intermediate and nuclear expression of DAF-16 (Fig. 5b). This indicated that the concentration of microplastic exposure can mitigate the effect. In contrast to our results, *Leon et al* observed a stress response with *C. elegans* exposure to 100 nm PS microplastics at a concentration of 10 mg/L[44]. This may be due to differences in the size of the tested plastic particles. The stress response observed in *Leon et al* was with microplastics of the same composition, polystyrene, but with a smaller diameter (100 nm) and at 10,000 x greater concentration than in this study. Indeed, we observed that 1 µm microplastics at our higher, 1 mg/L, concentration elicited a DAF-16 stress response. This indicates that a stress response via DAF-16 may be concentration-dependent. While we did not see a DAF-16-mediated stress response with our primary level of microplastic exposure (1 μM/L) alone, a significant response was elicited when DBP delivery was mediated with this same concentration of microplastics. Interestingly, *Leon et al* observed that with the removal of worms from microplastic exposure, the microplastics left the worm's body, and the DAF-16 stress response reduced. We did not test for the effect of DAF-16 localization with the removal of microplastic exposure from *C. elegans*, but it would be interesting to ask if the microplastic-mediated DBP stress response via DAF-16 would be maintained, as the microplastics might clear the worm body, while the DBP is absorbed.

*Exposure to polystyrene microplastics and microplastic DBP mixtures reduces life span.*

In some cases, the activation of a DAF-16 stress response is associated with an extended life span[59,62–64], and this response can be tissue-specific, with greater extension of lifespan associated with intestinal-specific DAF-16 activation[65]. Increased longevity induced by stress events is associated with the insulin/insulin-like growth factor (IGF-1) receptor signaling pathway, or diet, amongst other pathways[64]. In particular, due to the presence of microplastic accumulation in the gut of the worm (Fig. 2a), and studies indicating intestine-specific stress due to microplastic exposure[56], we wondered if the presence of microplastics may reduce nutrient intake and increase lifespan.

We next addressed whether microplastics or microplastic-mediated DBP exposure would alter *C. elegans* lifespan. Short-term exposure (48 hours) of *C. elegans* to 1 μm microplastic (1 μg/L) did not change the lifespan of animals compared to the control (Fig. 6a). Interestingly, when nematodes were exposed to the same conditions, but without removal after 48 hours, there was a significant reduction in lifespan ($p < 0.0001$) (Fig. 6a, S3a,b). This reduction in lifespan was equal to what was observed with continuous exposure to 1000 x more concentrated microplastics (1 mg/L) with continuous exposure (Fig. 6a). The lifespan of animals was further reduced when exposed to microplastics that mediated the delivery of 0.1 M DBP ($p = 0.00357$) (Fig. 6b, S3c). This reduction in lifespan was not observed under the control conditions of microplastics containing DMSO, or with exposure to DBP or DMSO alone (Fig. 6b, S3c). Additionally, the reduction in lifespan with microplastic-mediated DBP exposure appears to be dependent on the concentration of DBP, as the lowest concentration of DBP (100 μM) used in this study did not lead to a further reduction in lifespan as compared to microplastic exposure alone (Fig. S3c).

There are conflicting studies on the effect of microplastic exposure on *C. elegans* lifespan. In one study, polystyrene microplastics initiated a DAF-16 stress response that was not associated with a change in lifespan in *C. elegans*, and this was speculated to be due to a counteracting mechanism[44]. Other reports were consistent with our results, showing reduced lifespan with exposure to polystyrene microplastics[39]. Furthermore, our work shows that microplastic-mediated delivery of DBP further reduced lifespan. Additional reports examined the effects of leachates from plastic[66], phthalates[67,68], and other types of MP exposure on life span, all leading to reduced longevity.

This work shows that microplastic-mediated delivery of plastic-containing compounds, such as DBP, enhances the physiological defects observed with microplastic exposure alone. The exposure level of microplastics, the time of exposure, and the concentration of the absorbed chemicals influence the defects observed. Overall, our results show that chronic microplastic exposure has detrimental effects on reproduction and reduces lifespan. These defects are further exacerbated by the co-exposure of microplastics and DBP. Microplastics mediate the delivery of DBP in *C. elegans*, further decreasing fertility and lifespan as well as leading to a stress response via DAF-16 activation. Further studies are needed to identify other mechanisms involved in the toxicity due to microplastic-mediated exposure to DBP. The worsened physiological defects seen with co-exposure to phthalate-containing microplastics demonstrate that microplastics can leach contents into an organism after ingestion. This highlights the risks of microplastics releasing the additives they already contain and/or chemicals that they have absorbed from the environment.

### Methods

## Characterization of microplastics

Polystyrene microplastic (Poly Sciences, 19518-500) size and surface were characterized by Scanning Electron Microscopy (JEOL JSM-6010 PLUS/LA). Samples were sputter-coated with 2 nm gold particles prior to imaging (Quorum Technologies EMS150T ES). Microplastic diameter was measured using the measuring tool in the JOEL associated software.

### Preparation of microplastic and chemical mixtures

Microplastics were suspended in *E. coli (*strain OP50), seeded on Nematode Growth Media (NGM) plates (Teknova, N1105) and allowed to dry overnight before adding nematodes to the plates for exposure. To prepare microplastic *E. coli* suspensions, microplastics were suspended in a 1% solution with M9 buffer, then diluted into *E. coli* OP50. At each dilution step, the solution was sonicated for 15 minutes (Emerson Branson 3800). Wide-boar tips were used to transfer liquid. Dry 1 μM polystyrene microspheres (Poly Sciences, 19518-500) were used for all experiments unless otherwise noted. Di-butyl phthalate (DBP, Sigma, 524980) was diluted into 100% DMSO (Sigma, D8418) at 0.1 M concentration. Additional dilutions were made from the 0.1 M stock into water. DBP polystyrene microplastic mixtures were prepared by adding DBP (either 0.1 M, 3.7 mM, or 100 μM concentration) to dry polystyrene microplastics, to create a 1% solution, which was incubated overnight, on a rotating rack, in the dark, at room temperature (19–22°C). DMSO polystyrene microplastic controls were prepared by adding 100% DMSO, 3.7% DMSO, or 0.0001% DMSO to dry polystyrene microplastics at 1%. Polystyrene microplastics were diluted into *E. coli* OP50 to a final concentration of either 1 μg/L or 1 mg/L.

### Maintenance of C. elegans

*Caenorhabditis elegans (C. elegans)* stains, N2 (wild type) and TJ356 (zIs356 [daf-16p::daf-16a/b::GFP + rol-6(su1006)]) that were used in this study were provided by the Caenorhabditis Genome Center. Worms were grown on Nematode Growth Media Agarose (NGM) plates seeded with *E. coli* OP50 at 20°C.

### Exposure design

Bleach-synchronized L1 larvae were exposed to *E. coli* OP50 containing 1 μm polystyrene microplastics at either 1 μg/L or 1 mg/L, or polystyrene microplastics soaked in DBP (100 μM, 3.7 mM or 0.1 M) or DMSO (0.1%, 3.7%, or 100%) for 24 hours, or DBP or DMSO added directly to the OP50 *E. coli* alone on NGM plates made within 48 hours. DBP and DMSO only control concentrations were determined by the amount of solution carried into the final *E. coli* OP50, after dilution from the prepared 1% polystyrene microplastics. From a 1% polystyrene microplastic in 0.1 M DBP solution that was diluted 1:10,000 into *E. coli* OP50 for a final concentration of 1 μg/L microplastic, would have an associated 10 μM DBP control in *E. coli* OP50. L1s were exposed for 48 hours and then moved to *E. coli* OP50 plates for short-term exposure and maintained on prepared plates until the end points for experimental conditions. At least 2 biological replicates were performed for each assay.

## Visualizing microplastics in C. elegans

To visualize microplastic distribution and location, L1 bleach-synchronized worms were exposed to 1 μm internally dyed Dragon Green polystyrene microspheres (Bangs Laboratories Inc., FS03F). L1 synchronized larvae were exposed to *E coli* OP50 and 1 μM/L and 1 mM/L Dragon Green microplastics suspended in *E. coli* OP50 and seeded on NGM plates. Worms were paralyzed in levamisole on an agarose pad before imaging at each life stage.

### Visualizing Nile Red in C. elegans

To assess if chemical compounds absorbed by microplastic beads could transfer into the worm's body after ingesting the chemically soaked beads, we fed L1 synchronized *C. elegans* microplastic beads soaked in 1 mg/L Nile Red in methanol (Sigma Aldrich, 19123). The beads were rocked for 1 hour and left to dry overnight, before being suspended in a 1% solution in M9 buffer. The 1% M9 solutions were diluted to either 1 μg/L or 1 mg/L into OP50 *E. coli* before seeding on NGM plates and left to dry overnight.

### Lifespan Assay

Lifespan assays were performed at 20°C as previously described. Approximately 50 synchronized L1 larvae were placed onto corresponding treatment conditions for 48 hours and then moved to new plates containing the same treatment, with 100 μM 5'-fluorordeoxyuridine. Worms were counted as dead or alive every day until the number of live worms reached zero.

## Evaluation of reproductive toxicity

To assay the total number of eggs laid per worm and the percentage hatched, bleach-synchronized L1 larvae were seeded onto each condition. 48 hours after seeding, L4 larval worms were then singled out onto respective plates. The following day, L4 singling is repeated from the previous day's plate, and both plates are kept. After 24 hours, L1s and embryos are counted from the first day of singling, then repeated every day, counting the L4-free plate, until the worm stops laying (around 5–6 days).

## Imaging and microscopy

Fluorescent and bright field images were all taken using an EVOS M5000 (Invitrogen). For live imaging (Dragon-Green polystyrene microplastic localization, Nile Red soaked polystyrene microplastics transfer, and DAF-16-GFP localization), worms were transferred to an agarose pad on a slide and immobilized with levamisole. All image processing and analysis was done with ImageJ software.

## Activation of stress response

To determine if microplastic and chemical exposure lead to stress activation, the strain TJ356, expressing DAF-16 fused to GFP was exposed to polystyrene microplastics alone, and with DBP or DMSO, and DBP and DMSO alone. L1 synchronized larvae were placed on each condition and after 72 hours of exposure, imaged live. Live imaging was completed within 5 minutes of paralysis for each strain. Each nematode was classified by the localization of DAF-16::GFP expression (cytoplasmic, intermediate or nuclear)[61,62].

## Statistical Analysis

Statistical analysis and figure design were done with GraphPad Prism 8 (GraphPad Software, CA, USA). Data were checked for normality between treatments and statistical analysis was done using Kruskal-Wallis followed by Dunn's post hoc test (microplastic diameter, brood size, polystyrene microplastic exposure). A t-test (two-tailed) was done to analyze control vs. Nile Red data. Experiments were all carried out with a minimum of 2–3 biological replicates (N) and number of total worms (n) examined that are used in statistical analysis as described.

### Declarations

#### Acknowledgements

We thank V. Culotta for valuable discussions and advice on the development and design of experiments. Many undergraduate student researchers have played a part in moving this project forward, and we thank them for their time and valuable input. We also thank St. Mary's University for its continued support, including funds from the Department of Biological Sciences, the Benjamin F. Biaggini Endowment, and San Antonio Area Foundation. *C. elegans* strains were provided by the CGC, which is funded by the NIH Office of Research Infrastructure Programs (P40 OD010440). Research funding was provided by the National Institutes of Health, NIGMS (R16GM150406). C. Maldonado, P. Garcia, and M. Flores were funded by NIH grants (T34GM00873 and T34GM149455). Alyssa was funded by an NIH grant (R25GM102783).

#### Author contributions

C.M. planned and evaluated results on brood size and longevity, and wrote the paper. D.M. provided invaluable technical help throughout, performed experiments, and evaluated results. P.G. planned and carried out experiments on brood size and longevity. M.G. planned and performed fluorescent analysis. M.F. planned experiments and performed chemical transfer assays. A.F. and R.L.P. planned and carried out experiments to validate plastic particles using SEM. J.C.H. conceptualized, planned, trained students, executed some of the experiments, evaluated all results, and wrote the paper. All authors provided feedback on the manuscript.

#### Competing interests

We have no competing interests to disclose.

#### Materials & Correspondence

All requests and correspondence should be addressed to Jennifer C. Harr, PhD.

#### Data availability statement

The datasets generated during and/or analyzed during the current study are available from the corresponding author on reasonable request.

## Figures

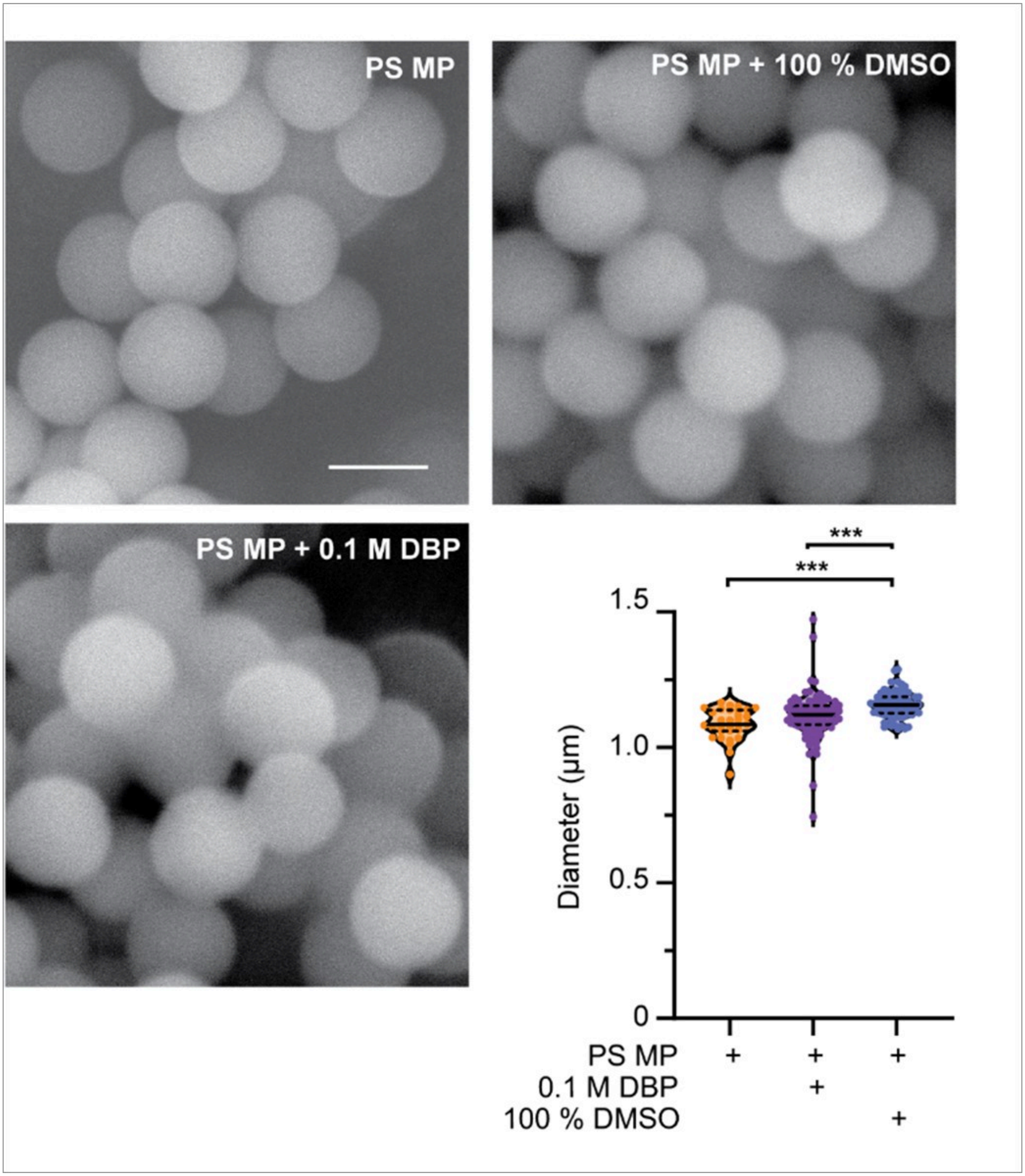

**Figure 1**

Characterization of polystyrene (PS) microplastics (MP) before and after 24-hour exposure to di-butyl phthalate (DBP) and DMSO. Representative SEM images of 1 µM PS MPs to verify their size and surface structure before and after exposure to 0.1 M DBP and 100 % DMSO. Error bar is 1 µM. Quantification of PS MP size before and after 24-hour exposure to 0.1 M DBP and 100 % DMSO. n= 31, 104, 81, ***p ≤ 0.0001.

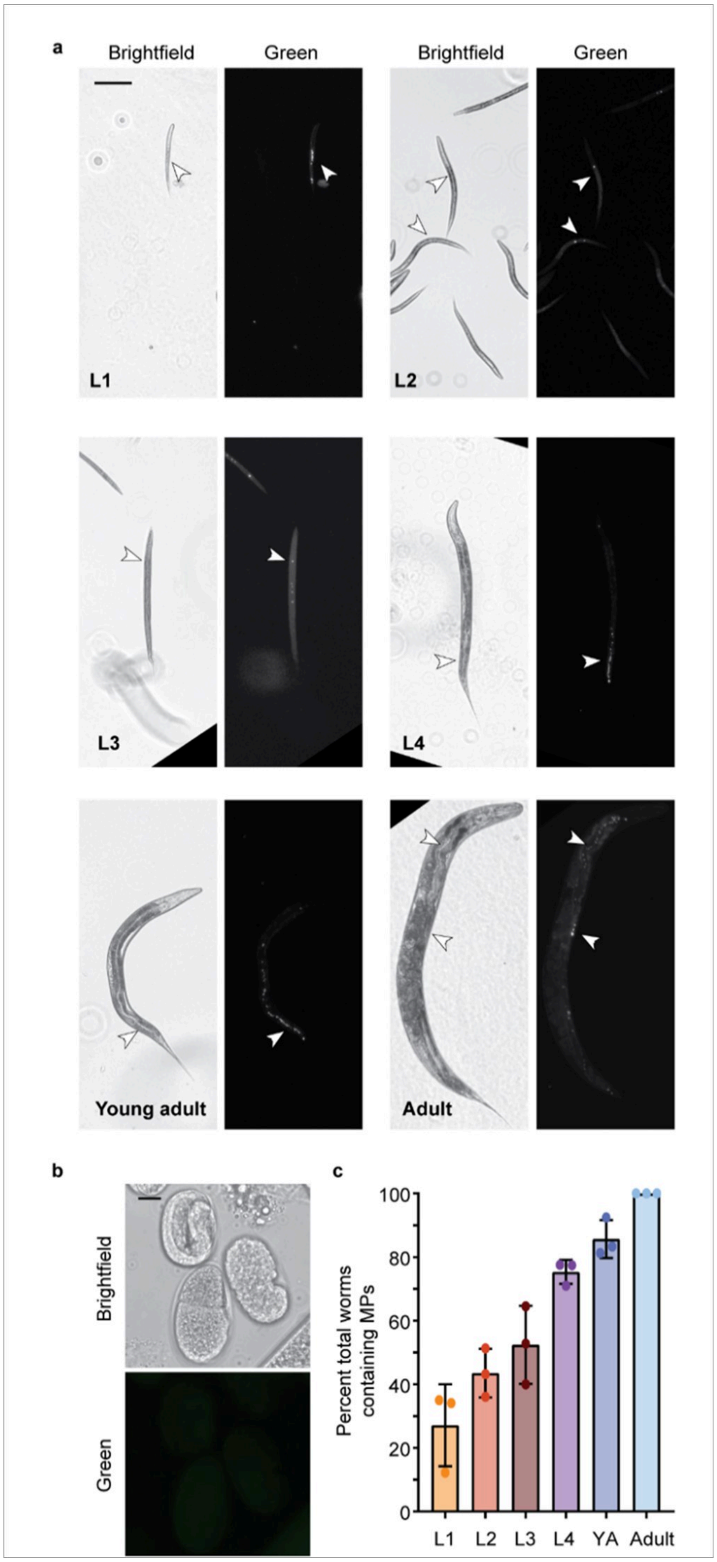

### Figure 2

1 µm Dragon Green Polystyrene (PS) Microplastics (MPs) are ingested by *C. elegans*. MPs are ingested by *C. elegans* at all larval stages. a) Representative images of L1-Adult *C. elegans* after continuous exposure to 1 µg/L Dragon Green 1 µm PS MP particles. Scale bar=100 µm. b) Representative image of *C. elegans* eggs from an adult worm that was continuously exposed to Dragon Green PS MPs. Scale bar=10 μm. c) Quantification of the number of worms containing MPs at each larval stage (L1- Adult). YA, young adult. N=3, n=173, 247, 199, 111, 111, 105.

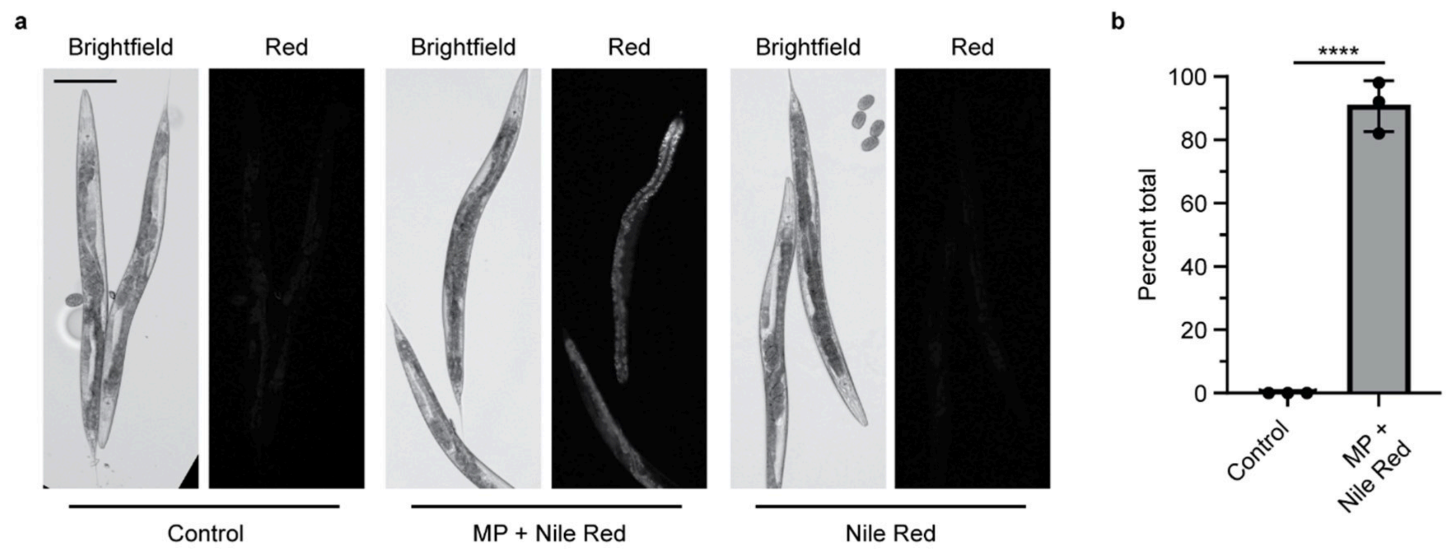

### Figure 3

**Microplastics soaked in Nile Red act as a carrier to deliver Nile Red into *C. elegans* after ingestion.** a) Representative images of adult *C. elegans* fed *E. coli* OP50 with and without Nile Red-stained microplastics (MPs). Control = *E. coli* OP50 only. Scale bar = 100 μm. b) Quantification of the presence of Nile Red inside *C. elegans* after ingestion of Nile Red-stained MPs, N = 3, n = 50, 50, 30. ***p<0.00001.

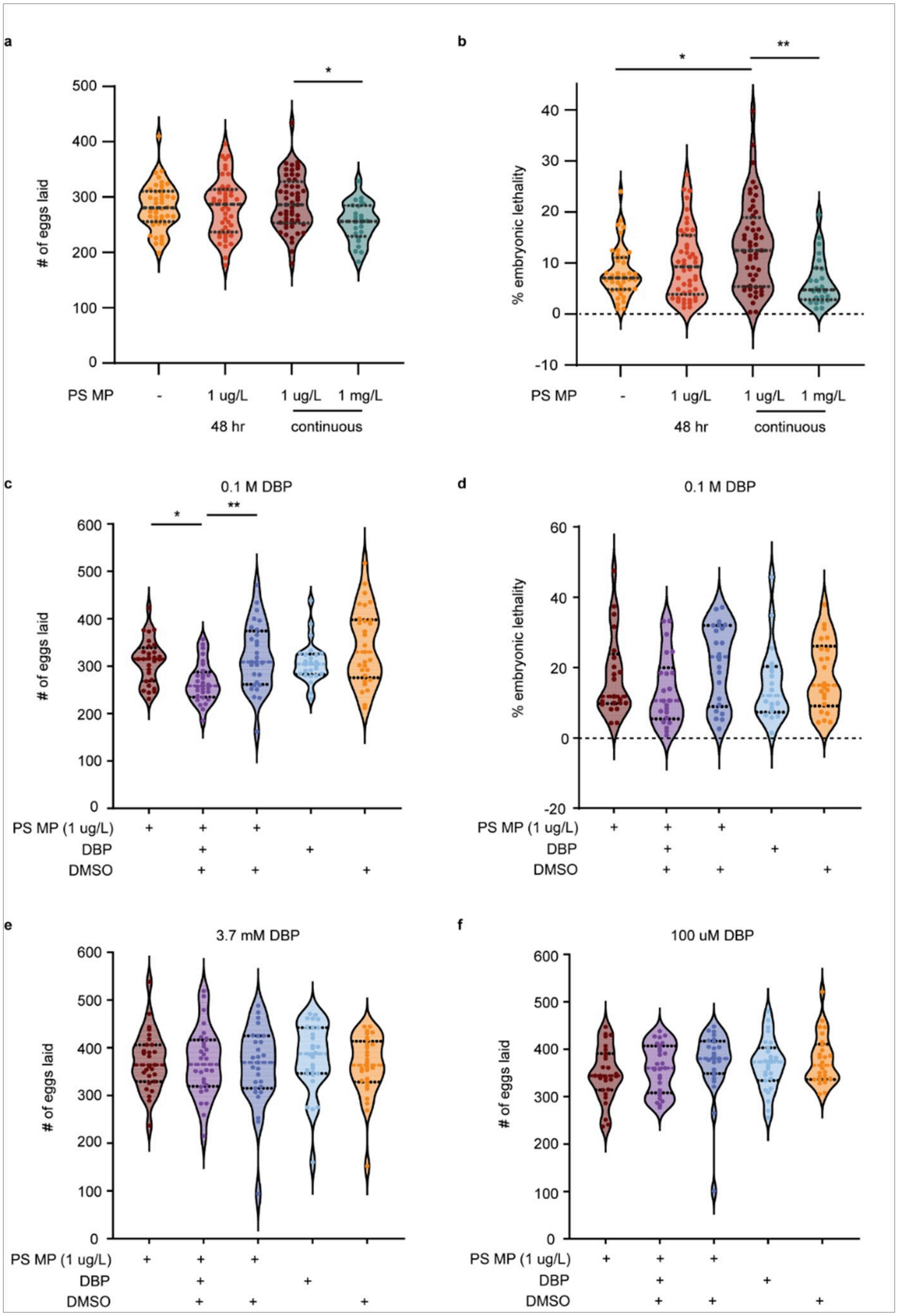

### Figure 4

Total eggs laid, number of fertilized and unfertilized eggs, and embryonic lethality of *C. elegans* exposed to polystyrene (PS) microplastics (MP) and/or containing DBP. a) Number of eggs laid per nematode with *E. coli* OP50 only, 48-hour exposure to PS MP or continuous PS MP exposure. N=8, n=43, 46, 52. b) Percent embryonic lethality. c) Total number of eggs with exposure to continuous PS MP with and without 0.1 M DBP. N= 4, n= 28, 28, 28, 25, 29. d) Percent embryonic lethality. e) Total number of eggs with exposure to continuous PS MP with and without 3.7 mM DBP. N=4, n=27, 28, 27, 28, 28. f) Total number of eggs with exposure to continuous PS MP with and without 100 μM DBP. N=3, n=27, 27, 25, 27, 26. Violin plots show the mean with the standard deviation. Kruskal-Wallis was used for statistical analysis, using Dunn's as a post hoc test (*p ≤ 0.05; **p ≤ 0.01).

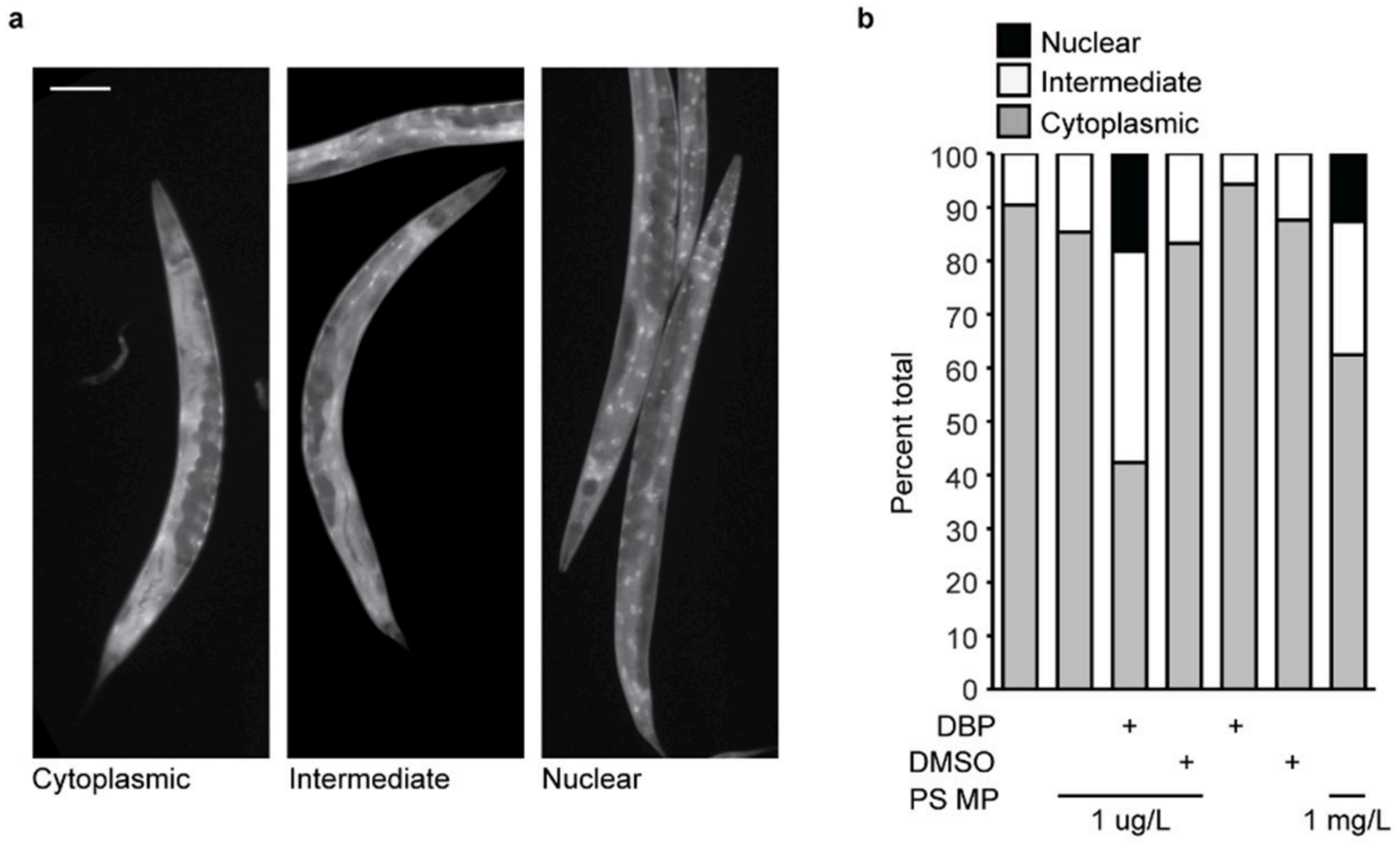

### Figure 5

Stress response via DAF-16 in *C. elegans* after exposure to polystyrene (PS) microplastic (MPs) and DBP. a) Representative images of DAF-16-GFP expression in adult *C. elegans*, showing cytoplasmic (left), intermediary (center), and nuclear localization (right). Scale bar = 100 μm. b) Quantification of DAF-16-GFP localization in *C. elegans* with exposure to their regular E. coli diet only, with 1 μg/L PS MP, 1 μg/L PS MP and DBP, 1 μg/L PS MP and DMSO (vehicle control), DBP alone, and DMSO alone. N=2, n=53, 65, 170, 149, 140, 129, 43.

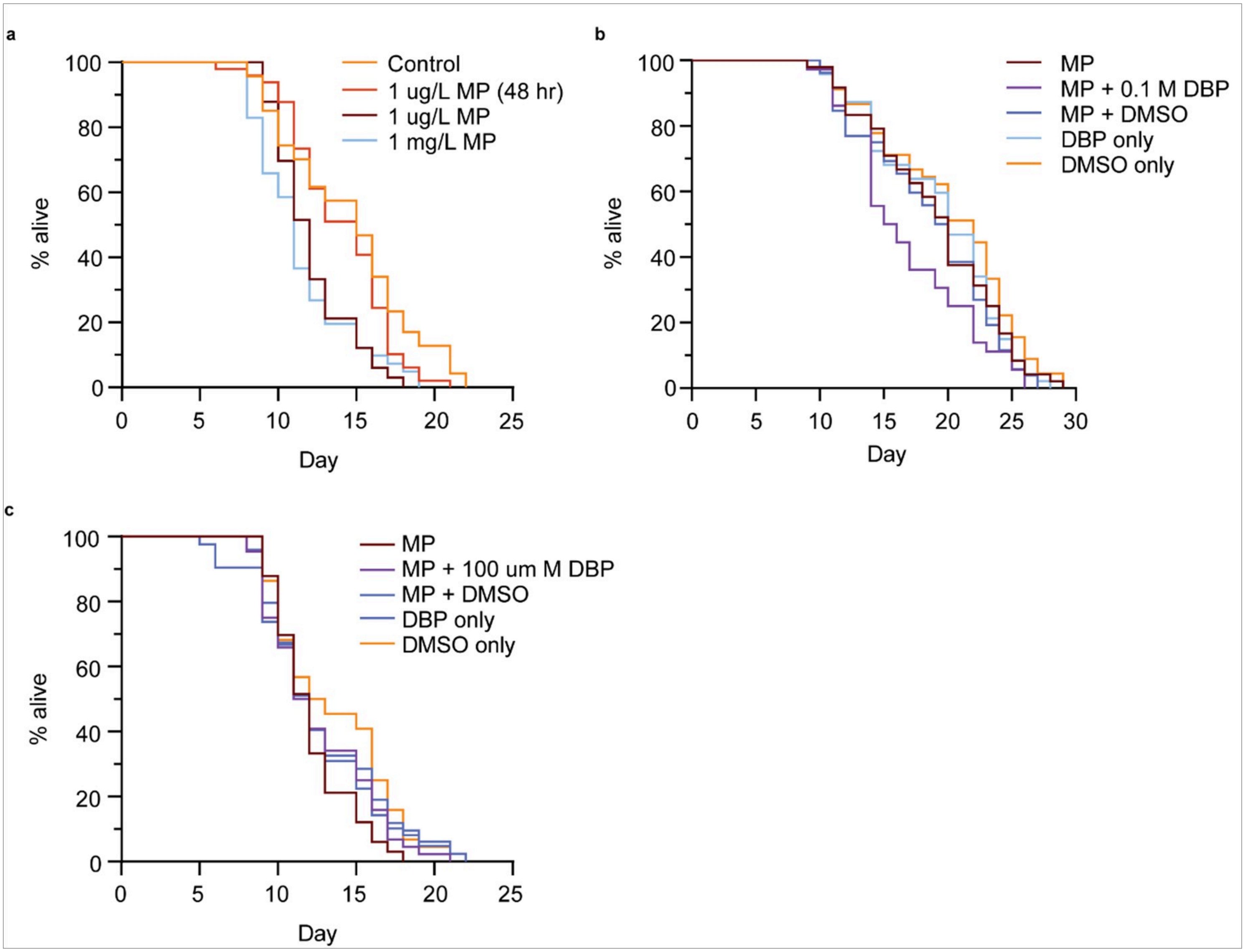

### Figure 6

Lifespan of *C. elegans* exposed to polystyrene (PS) microplastic (MPs) containing different concentrations of MPs or MPs that had been soaked in different concentrations of DBP (0.1 M or 100 μM). a) Survival assay for nematodes exposed to control (no MPs), 1 μg/L PS-MPs for 48 hours, 1 μg/L or 1 mg/L PS-MPs continuously. n=47, 49, 33, 41. p<0.0001. b) Survival assay for N2 nematodes continuously exposed to 1 μg/L PS MPs, with and without being soaked in 0.1 M DBP or vehicle alone (100 % DMSO) for 24 hours. n=48, 36, 52, 47, 45. p=0.00357. c) Survival assay for N2 nematodes continuously exposed to 1 μg/L PS-MPs, with and without being soaked in 100 μM DBP or vehicle alone (0.1 % DMSO) for 24 hours. n=33, 44, 42, 49, 44. Not significant, p=0.4. Log-rank (Mantel-Cox).

## Supplementary Files

This is a list of supplementary files associated with this preprint. Click to download.

- SupplementalFiguresMaldonado.docx
